# Green Synthesis of Silver Nanoparticles Using *Circaea lutetiana* Ethanolic Extract: Phytochemical Profiling, Characterization, and Antimicrobial Evaluation

**DOI:** 10.3390/ijms26125505

**Published:** 2025-06-08

**Authors:** Zhanar Iskakova, Akmaral Kozhantayeva, Aliya Temirbekova, Saule Mukhtubayeva, Gulmira Bissenova, Zhanar Tekebayeva, Kairtai Almagambetov, Yerbolat Tashenov, Zinigul Sarmurzina

**Affiliations:** 1 Research Institute of New Chemical Technologies, L.N. Gumilyov Eurasian National University, Satpayev Street 2, Astana 010000, Kazakhstan; iskakova_zhb@enu.kz; 2 Department of Chemistry, Faculty of Natural Sciences, L.N. Gumilyov Eurasian National University, Satpayev Street 2, Astana 010000, Kazakhstan; 3 Republican Collection of Microorganisms, Astana 010000, Kazakhstan; atemirbekova94@gmail.com (A.T.); zanartekebaeva@gmail.com (Z.T.); rcmkz@list.ru (K.A.); sarmurzina@list.ru (Z.S.); 4 Astana Botanical Garden, Astana 010008, Kazakhstan

**Keywords:** *Circaea lutetiana*, phytochemical screening, polyphenolic compounds, silver nanoparticles (AgNPs), nanoparticle characterization, antibacterial activity

## Abstract

In the current decade, the use of plant extracts for the green preparation of metal nanoparticles has garnered increasing attention due to their eco-friendliness, cost-effectiveness, and sustainability. In the current study, silver nanoparticles (AgNPs) were synthesized using the ethanolic extract of *Circaea lutetiana* for the first time. Thetotal flavonoid content (TFC) and total phenolic content (TPC)of the extract were analyzed by spectrophotometric methods. Fourier transform infrared (FT-IR) spectroscopy was employed to determine the functional groups involved in both the reduction and stabilization processes of AgNPs. The formation and optical properties of AgNPs were confirmed by Ultraviolet–Visible (UV–Vis) spectroscopy. The greenlysynthesized AgNPs were characterized by FT-IR, UV–Vis, X-ray diffraction (XRD), X-ray photoelectron spectroscopy (XPS), dynamic light scattering (DLS) and zeta potential analyses, transmission electron microscopy (TEM), and scanning electron microscopy (SEM). The results confirmed that the AgNPs were spherical in shape with an average size of approximately 3.8 nm and showed a good crystalline nature. Additionally, the AgNPs exhibited significant antimicrobial activity against both Gram-positive and Gram-negative bacteria, demonstrating their potential as green antimicrobial agents.

## 1. Introduction

Herbal plants play a crucial role in human health due to their bioactive phytochemicals, offering both medicinal and economic benefits. Before the advent of synthetic drugs, herbs were widely used to treat various ailments and continue to be valued in traditional medicine [1]. The *Onagraceae* family includes approximately650 species of trees, shrubs, and herbs distributed among 17 genera, and it is divided into two subfamilies: *Ludwigioideae*, mainly consisting of *Ludwigia* species, and *Onagroideae* [2,3]. One notable genus within this family is *Circaea* L., consisting of 12 species distributed across Asia, Europe, and Africa [1]. In Russia, several *Circaea* species are included in the Red Book across 23 regions [4]. In Kazakhstan, only two species—*Circaea alpina* L. and *Circaea lutetiana* L.—naturally occur in the thick forested areas ranging from the Altai Mountains to the Zhungarian Alatau [5,6]. Commonly known as Enchanter’s nightshade, *C. lutetiana* is a long-living herb with underground rhizomes that thrives in the damp, mixed forests of deciduous and coniferous trees across the Northern Hemisphere [7,8]. Traditionally, this plant has been valued in folk medicine for treating skin conditions, healing wounds, supporting urinary tract health, and relieving digestive issues like diarrhea and stomach pain [9,10,11]. It is also employed for conditions associated with inflammation [12].

Phytochemical investigations of *C. lutetiana* have revealed a rich and diverse composition, including polyphenolic compounds, tannins, and tocopherols. Granica et al. identified a total of fourteen polyphenolic compounds in the aerial parts of the plant, including phenolic acids like gallic acid and caffeic acid pentoside; three ellagitannins, with oenothein B being the most prominent; six flavonoids, such as apigenin glycosides, isoquercitrin, and astragalin; along with ellagic acid derivatives and one unidentified compound [10]. Additional studies have confirmed the presence of both C-glycosyl flavones—like vitexin and luteolin-6-C-glucoside—and O-glycosyl flavones, such as apigenin 7-O-glucoside and quercetin 3-O-glucoside [7,10]. Furthermore, the plant contains unique compounds like icariside B2, and its seeds have been explored as potential sources of fatty acids and tocopherols [7,10,12]. ICP-MS has also identified 41 mineral elements in different plant parts, with notably high levels of iron and beryllium in the roots [13].

Biological research has shown that *C. lutetiana* extracts exhibit strong antioxidant and anti-inflammatory effects [14]. Notably, the extract demonstrated potent free radical scavenging activity, with SC_50_ values of 4.0 ± 2.3 μg/mL for superoxide anions and less than 2 μg/mL for hydrogen peroxide—both outperforming vitamin C. The DPPH radical scavenging activity was measured at SC_50_ = 33.1 ± 3.1 μg/mL. These activities are largely attributed to oenothein B, the major bioactive component. Additionally, the extract inhibits hyaluronidase (IC_50_ = 13.3 ± 2.4 μg/mL, stronger than heparin) and lipoxygenase (IC_50_ = 44.7 ± 1.4 μg/mL, more potent than indomethacin) [12]. 

In recent years, there has been growing interest in eco-friendly methods for synthesizing nanomaterials [15]. Green synthesis, which involves using plant extracts, microorganisms, or other natural materials as reducing and stabilizing agents, has emerged as a safer and more sustainable alternative to conventional methods that often depend on toxic chemicals and produce hazardous by-products [16,17,18]. Rooted in the principles of green chemistry, this approach utilizes the rich phytochemical profile of medicinal plants, including polyphenols, flavonoids, tannins, and alkaloids, which act simultaneously as reducing agents and stabilizers during nanoparticle formation [19].

Silver nanoparticles (AgNPs) are particularly notable for their impressive range of biological activities. When produced using plant extracts, they have demonstrated strong antimicrobial, antioxidant, anticancer, and anti-inflammatory effects [16]. They are widely utilized in biomedical fields, including wound healing, targeted drug delivery, medical device coatings, and water purification [19,20]. Their modes of action include damaging microbial membranes, generating reactive oxygen species, and disrupting vital intracellular processes like DNA replication [19,21]. Notably, greenlysynthesized AgNPs tend to be more biocompatible and less toxic compared to chemically synthesized ones, enhancing their potential for clinical applications [20,22,23,24]. Several studies have confirmed that AgNPs produced using plant extracts like *Origanum vulgare* or *Berberis vulgaris* effectively inhibit the growth of both Gram-positive and Gram-negative bacteria [19]. Their enhanced bioactivity is largely attributed to the small size, largesurface area, and the presence of bioactive phytochemicals on the nanoparticle surface [21]. In addition to silver nanoparticles, green synthesis strategies have also been successfully applied to other metal oxide nanoparticles, such as copper oxide (CuO) and zinc oxide (ZnO). For instance, CuO NPs synthesized using a *Morinda citrifolia* leaf extract exhibited excellent optical switching and third-order nonlinear optical properties, demonstrating the versatility of plant-mediated nanomaterials in photonic applications [25]. Similarly, ZnO NPs prepared via green routes have shown significant antimicrobial and antioxidant activities, further supporting the functional potential of biogenic metal oxide nanostructures [26].

*C. lutetiana*, due to its rich phytochemical profile, including ellagitannins (notably oenothein B), flavonoid glycosides, and phenolic acids [7], offers a promising platform for green synthesis. These compounds naturally act as both reducing and stabilizing agents during the formation of silver nanoparticles, making it possible to produce nanomaterials that are both bioactive and biocompatible. Although interest in green nanotechnology has been steadily increasing, *Circaea lutetiana* has not yet been investigated as a potential source for nanoparticle synthesis. This study, therefore, set out to explore the use of a *C. lutetiana* extract as a natural reducing and stabilizing agent in the green synthesis of silver nanoparticles.

## 2. Results

### 2.1. Phytochemical Composition of Circaea lutetiana Extracts

#### 2.1.1. Extraction Yield Analysis

The yield of extractable compounds from the aerial parts of *C. lutetiana* was determined following Soxhlet extraction using ethanol and ethyl acetate as solvents. After a 5 h extraction process, the dry residues were carefully weighed, and the extraction yield was calculated based on the dry weight of the starting plant material. The ethanol-based extraction produced a yield of 15.4 ± 0.6% (*w*/*w*), whereas the ethyl acetate extract showed a lower yield of 7.3 ± 0.4% (*w*/*w*).

#### 2.1.2. Total Phenolic and Flavonoid Contents (TPC and TFC)

As part of the phytochemical assessment of *C. lutetiana*, the total phenolic content (TPC) and total flavonoid content (TFC) were determined in ethanol and ethyl acetate extracts using standard spectrophotometric techniques. The analysis revealed notable differences in the concentrations of bioactive compounds depending on the solvent employed.

The total phenolic content (TPC) was determined using the Folin–Ciocalteu method and expressed as milligrams of gallic acid equivalents per gram of dry matter (mg GAEs/g DM). The ethanol extract exhibited a TPC of 89.16 ± 1.15 mg GAEs/g DM, whereas the ethyl acetate extract showed a lower content of 14.23 ± 0.85 mg GAEs/g DM (Table 1). The observed results indicate that ethanol is more effective than ethyl acetate at extracting phenolic compounds from the plant matrix.

Similarly, the total flavonoid content was assessed using the aluminum chloride colorimetric method and reported as milligrams of quercetin equivalents per gram of dry matter (mg QEs/g DM). The ethanol extract demonstrated a TFC value of 8.06 ± 0.32 mg QEs/g DM, while the ethyl acetate extract showed a slightly lower value of 7.24 ± 0.18 mg QEs/g DM.

#### 2.1.3. HPLC-UV-ESI/MS Analysis

The HPLC-UV-ESI/MS analysis of *C. lutetiana* extracts identified and quantified phenolic acids and flavonoids, revealing distinct differences between the ethanol (Cir-EtOH) and ethyl acetate (Cir-EtOAc) extracts (Figure S1). In general, ethanol extraction yielded higher concentrations of phenolic compounds, whereas ethyl acetate was selective for specific non-glycosylated flavonoids. Among the phenolic acids, gallic acid was the most abundant, with a concentration of 15.1 mg/g in Cir-EtOH and 3.40 mg/g in Cir-EtOAc. p-Coumaric acid was also highly concentrated in Cir-EtOH (14.6 mg/g) but was not detected in the ethyl acetate extract, highlighting the role of solvent polarity in compound extraction (Table 2). Similarly, chlorogenic acid was found in substantial amounts (10.5 mg/g in ethanol), but was not detected in the ethyl acetate fraction. Caffeic acid was also detected in both extracts but was present at significantly higher levels in ethanol (9.80 mg/g) compared to ethyl acetate (0.34 mg/g), confirming that polar solvents are more effective for extracting hydroxycinnamic acids. Regarding flavonoids, rutin (1.20 mg/g) and quercetin 3-glucoside (0.10 mg/g) were exclusively found in the ethanol extract. In contrast, apigenin was only detected in the ethyl acetate extract (0.02 mg/g), suggesting that ethyl acetate is more effective at extracting non-glycosylated flavonoids.

#### 2.1.4. FT-IR Analysis of *Circaea lutetiana* Extracts

**Figure 1:**
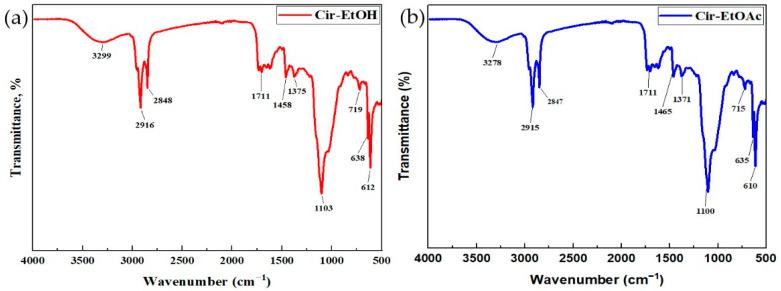
FT-IR spectra of the *C. lutetiana* (**a**) ethanol extract (Cir-EtOH) and (**b**) ethyl acetate extract (Cir-EtOAc).

FT-IR spectroscopy was employed to detect the functional groups of active compounds by analyzing their characteristic absorption peaks in the infrared spectrum [27]. The IR spectra of *C. lutetiana* extracts revealed multiple functional groups. Notably, broad absorption bands related to O–H stretching were detected at 3299 cm^−1^ in the ethanol extract and 3278 cm^−1^ in the ethyl acetate extract, indicating the presence of hydroxyl-containing compounds (Figure 1a,b). The prominent O–H absorption bands suggest the presence of phenolic compounds, including caffeic acid, gallic acid, chlorogenic acid, and various flavonoids. Additionally, C-H stretching was observed at 2916 cm^−1^ (Cir-EtOH) and 2915 cm^−1^ (Cir-EtoAc), corresponding to alkane (-CH_3_, -CH_2_-) and alcohol (-OH in acids) vibrations, suggesting the attachment of aromatic rings and alkyl groups or the presence offlavonoid glycosides (e.g., rutin and quercetin-3-glucoside) and chlorogenic acid. Further C-H stretching at 2848 cm^−1^ (Cir-EtOH) and 2847 cm^−1^ (Cir-EtoAc) supports the presence of aliphatic compounds. A distinct C=O stretching band observed at 1711 cm^−1^ in both the ethanol (Cir-EtOH) and ethyl acetate (Cir-EtOAc) extracts points to the presence of carbonyl-containing groups such as carboxylic acids, esters, and ketones. These functional groups are typically associated with phenolic acids like caffeic acid, ferulic acid, and *p*-coumaric acid [28,29].

The peaks at 1465 cm^−1^ (Cir-EtoAc) and 1458 cm^−1^ (Cir-EtOH) correspond to methyl (-CH_3_) and methylene (-CH_2_-) bending vibrations, suggesting alkyl side chains found in ferulic acid, rosmarinic acid, and flavonoids, which are typical interpenoids, lipids, and flavonoid glycosides. The peak values at 1371 cm^−1^ (Cir-EtoAc) and 1375 cm^−1^ (Cir-EtOH) show CH_3_ symmetric bending, indicating lipophilic substances or methyl-substituted flavonoids [28].

The C-O stretching vibrationsat 1100 cm^−1^ (Cir-EtoAc) and 1103 cm^−1^ (Cir-EtOH) indicate the presence of alcohols, esters, and ether bonds, confirming flavonoid glycosides and phenolic acids, consistent with the HPLC resultsthat identifiedrutin, quercetin 3-glucoside, chlorogenic acid, and rosmarinic acid [30,31].

### 2.2. Green Synthesis and Characterization of Silver Nanoparticles

#### 2.2.1. Synthesis and UV–Vis Spectroscopic Confirmation

The formation of AgNPs using the *C. lutetiana* ethanol extract follows a typical green synthesis pathway. The process begins with the reduction of Ag^+^ by phenolic compounds, leading to the nucleation of silver particles. These particles then grow, and their size is controlled by the capping effect of the phytochemicals. This stabilization is crucial in preventing aggregation, ensuring a uniform size distribution, as observed in the UV–Vis spectrum [32,33].

**Figure 2:**
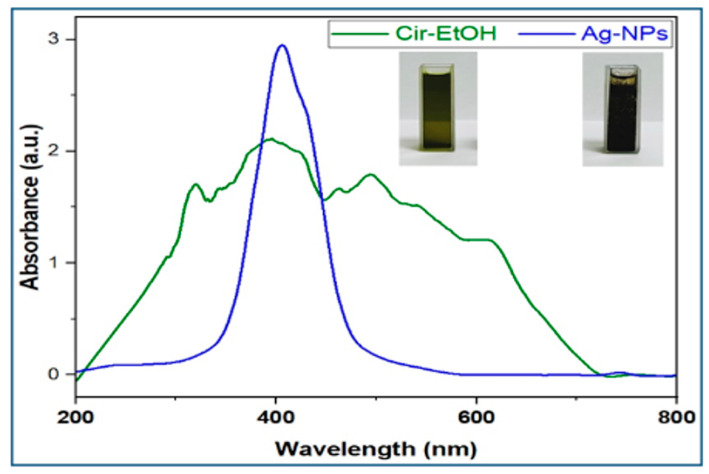
UV–Vis spectra of the *C. lutetiana* ethanol extract and synthesized AgNPs.

UV–visible spectroscopy served as a key tool to verify the successful synthesis of silver nanoparticles (AgNPs). The absorption spectrum of the ethanol extract of *C. lutetiana* shows a broad peak between 250 and 700 nm (Figure 2, green line), which is characteristic of the diverse phytochemicals present, including flavonoids and phenolic acids.

In comparison, the UV–Vis spectrum of the synthesized AgNPs (Figure 2, blue line) displays a clear and sharp surface plasmon resonance (SPR) peak centered at approximately 408 nm. This SPR absorption band is characteristic of silver nanoparticles and aligns with previous literature reports, confirming the successful reduction of Ag^+^ to metallic silver (Ag^0^) [34,35]. The sharpness of the peak and the absence of additional peaks suggest that the nanoparticles are stable, well-dispersed, and free from significant aggregation. The observed color change from dark green to dark brown during synthesis indicates the effective formation of AgNPs.

#### 2.2.2. Structural and Morphological Features of AgNPs

##### FT-IR Analysis of the Synthesized AgNPs

**Figure 3:**
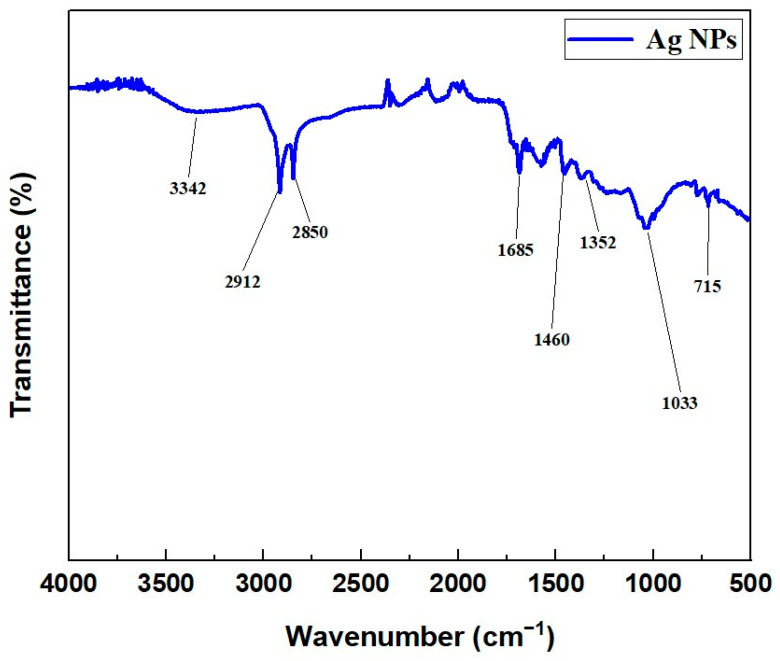
FT-IR spectrum of AgNPs prepared using the *C. lutetiana* ethanol extract.

Fourier transform infrared (FT-IR) spectroscopy was employed to identify the functional groups participating in the biosynthesis and stabilization of AgNPs. The FT-IR spectrum of silver nanoparticles synthesized with the ethanol extract of *C. lutetiana* is presented in Figure 3.

The FT-IR spectrum revealed a broad absorption band around 3342 cm^−1^, indicative of O–H stretching vibrations, suggesting the presence of hydroxyl and phenolic groups. Aliphatic C–H stretching was evident from the bands at 2912 and 2850 cm^−1^. A distinct signal near 1685 cm^−1^ was attributed to aromatic C=C stretching. Additional peaks within the 1352–1460 cm^−1^ range were linked to CH_2_ and CH_3_ bending vibrations, while the band observed around 1033 cm^−1^ was associated with C–O stretching. A weaker band appearing at approximately 715 cm^−1^ may correspond to Ag–O or Ag–N bonds.

##### XPS Surface Analysis

**Figure 4:**
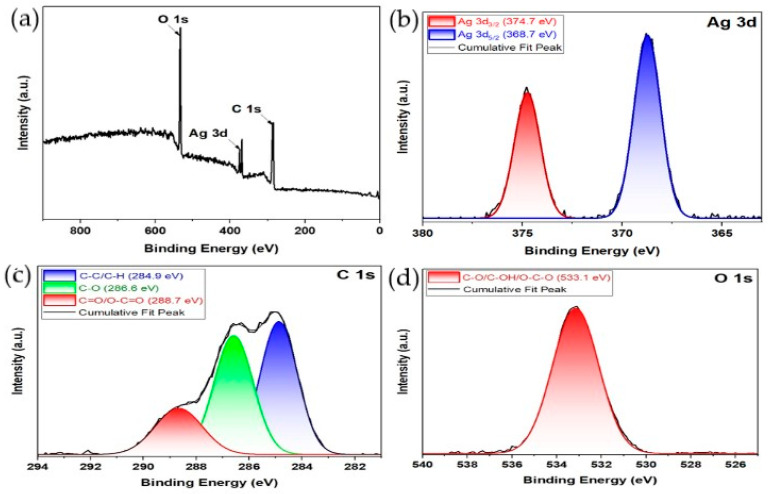
XPS spectra of green-synthesized AgNPs: (**a**) survey spectrum and high-resolution (**b**) Ag 3d, (**c**) C 1s, and (**d**) O 1s spectra.

X-ray photoelectron spectroscopy (XPS) was conducted to analyze the surface composition and identify the chemical states of the synthesized silver nanoparticles (AgNPs). The survey spectrum (Figure 4a) revealed the presence of silver (Ag), carbon (C), and oxygen (O), indicating the coexistence of metallic and organic components on the nanoparticle surface.

A high-resolution analysis of the Ag 3d region (Figure 4b) showed two peaks at 368.7 eV and 374.7 eV, corresponding to Ag 3d_5_/_2_ and Ag 3d_3_/_2_, respectively. The observed spin-orbit splitting of ~6.0 eV is characteristic of metallic silver (Ag^0^).

The C 1s spectrum (Figure 4c) revealed three distinct peaks at 284.9 eV, 286.6 eV, and 288.7 eV, corresponding to the C–C/C–H, C–O, and O–C=O functional groups, respectively.

The O 1s spectrum (Figure 4d) displayed a peak centered at 533.1 eV, associated with C–O, C–OH, or O–C=O environments.

##### XRD Crystallographic Analysis

**Figure 5:**
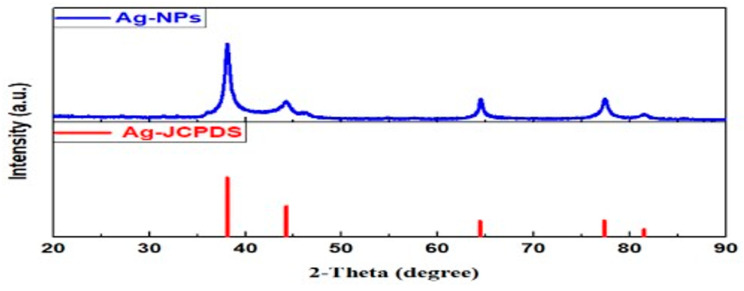
XRD patterns of AgNPs.

The XRD analysis verified that the silver nanoparticles synthesized using the *C. lutetiana* ethanol extract exhibit a face-centered cubic (fcc) crystalline structure. As illustrated in Figure 5, the diffraction peaks at 2θ values of 38.1°, 44.3°, 64.5°, and 77.4° correspond to the (111), (200), (220), and (311) planes of metallic silver. These reflections align with the standard JCPDS file No. 04-0783, confirming the crystalline nature of the greenlysynthesized AgNPs.

The average crystallite size was estimated to be 21–25 nm using the Scherrer Equation (1):

(1) $$D=\frac{K\lambda }{\beta cos\theta }$$

where *K* is the shape factor (0.9), λ is the X-ray wavelength (0.15406 nm), β is the FWHM in radians, and θ is the Bragg angle.

##### DLS and Zeta Potential Analyses

To assess the hydrodynamic diameter and surface charge of silver nanoparticles synthesized with the *C. lutetiana* ethanol extract, dynamic light scattering (DLS) and zeta potential analyses were performed.

**Figure 6:**
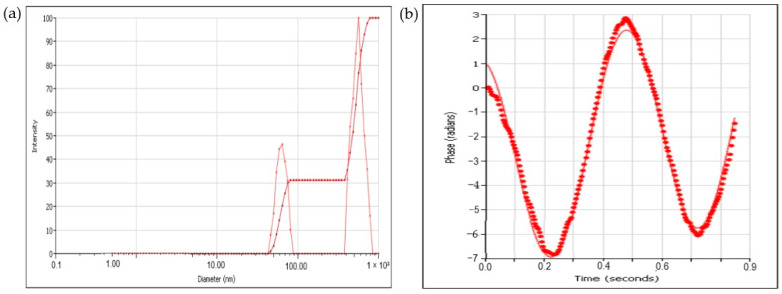
(**a**) Dynamic light scattering (DLS) analysis and (**b**) zeta potential distribution of the synthesized silver nanoparticles.

As shown in Figure 6a, the DLS analysis revealed a multimodal size distribution, with an average hydrodynamic diameter of approximately 192.36 nm by intensity and approximately 56.23 nm by number. The polydispersity index (PDI) was 0.529, suggesting a moderate degree of size variation among the nanoparticles.

Zeta potential values ranged from –25.62 to –28.19 mV (Figure 6b), suggesting the moderate electrostatic stability of the colloidal AgNPs in aqueous suspension.

##### TEM and SEM-EDX Imaging

**Figure 7:**
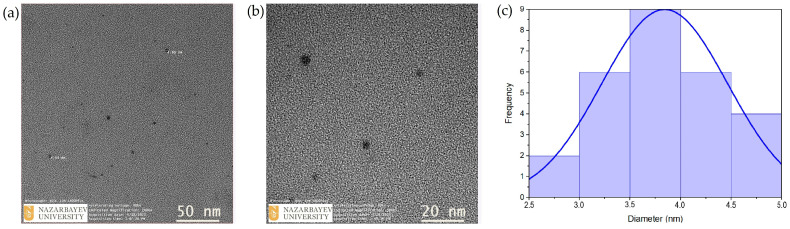
(**a**,**b**) Transmission electron microscopy (TEM) images of AgNPs at different magnifications; (**c**) particle size distribution of AgNPs.

Transmission electron microscopy (TEM) showed that the green-synthesized AgNPs were spherical in shape, well-dispersed, and exhibited minimal aggregation. The particle sizes ranged from 2.70 to 4.95 nm, with an average diameter of 3.84 ± 0.62 nm (Figure 7a–c).

**Figure 8:**
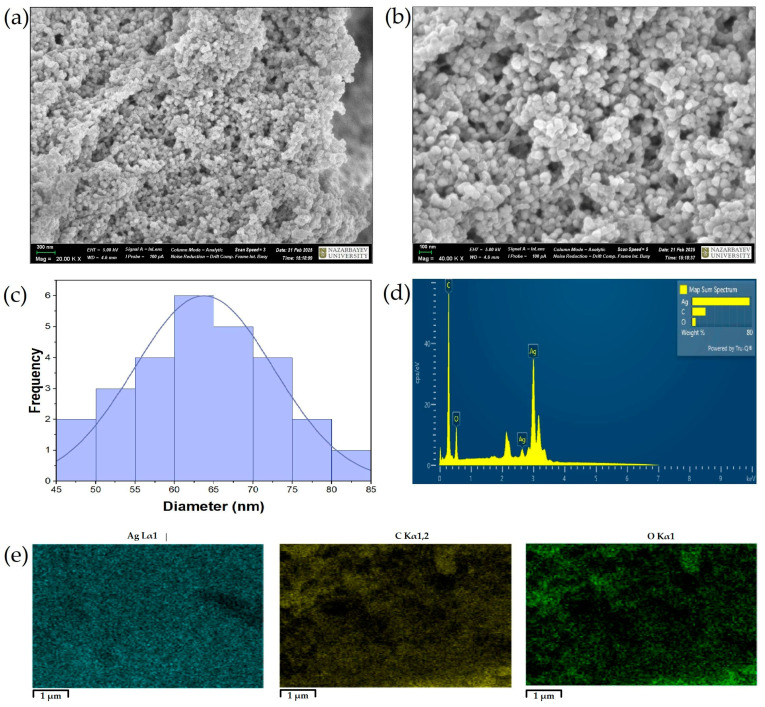
(**a**,**b**) SEM images of AgNPs at different magnifications; (**c**) particle size distribution histogram; (**d**) EDX spectrum showing elemental composition; and (**e**) elemental maps of Ag, C, and O.

Scanning electron microscopy (SEM) provided additional surface information. At 20.00 KX and 40.00 KX magnifications (Figure 8a,b), the particles appeared mostly spherical with moderate aggregation and a fairly uniform morphology. The size distribution histogram (Figure 8c) from SEM images showed particle sizes primarily in the 50–80 nm range, with a modal value of approximately 65 nm.

EDX spectrum (Figure 8d) confirmed the elemental presence of silver (Ag) via a prominent peak at ~3 keV, alongside minor peaks for carbon (C) and oxygen (O). Elemental mapping (Figure 8e) further demonstrated a uniform spatial distribution of Ag, C, and O, indicating a homogenous surface composition.

### 2.3. Antibacterial Activity of the Extracts and AgNPs

This research assessed the antimicrobial properties of *C. lutetiana* extracts and their corresponding silver nanoparticles (AgNPs), aiming to elucidate how green synthesis enhances bioactivity compared to plant-derived compounds alone. The outcomes highlight the limited but selective potential of the ethanol extract and the significantly stronger antibacterial effects of the synthesized AgNPs (Table 3 and Table 4).

#### 2.3.1. Comparative Activity of *C. lutetiana* Extracts

**Figure 9:**
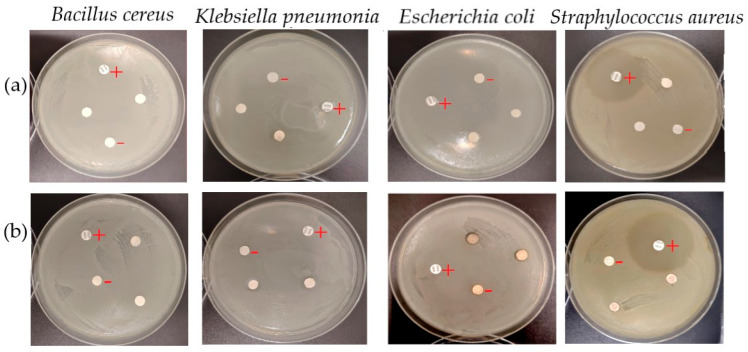
Disc diffusion assay showing the antibacterial activity of *C. lutetiana* extracts against selected bacterial strains. (**a**) Ethanolic extract; (**b**) ethyl acetate extract. (+) Positive control (penicillin); (−) negative control (DMSO).

Among the tested solvents, only the ethanolic extract (Cir-EtOH) exhibited weak antibacterial activity against selected strains, notably *Klebsiella pneumoniae* (2.54 ± 0.36 mm), *Escherichia coli* (1.07 ± 0.24 mm), and *Staphylococcus aureus* (0.59 ± 0.18 mm), whereas the ethyl acetate extract (Cir-EtOAc) demonstrated no inhibition against any tested microorganism (Table 3 and Figure 9).

According to the inhibition zone classification, the observed inhibition zone diameters fell into the “low activity” or “inactive” range. The largest inhibition zone was noted for *K. pneumoniae*, while *S. aureus* and *E. coli* showed minimal sensitivity. No inhibition was observed for *Bacillus cereus* or with the Cir-EtOAc extract.

#### 2.3.2. Antimicrobial Activity of Silver Nanoparticles

Silver nanoparticles (AgNPs) synthesized using the *C. lutetiana* ethanol extract demonstrated measurable antibacterial activity against selected bacterial strains when evaluated via both the disc diffusion method (DDM) and the agar well diffusion method (AWD). In the DDM assay, the inhibition zone for *Staphylococcus aureus* reached value of 2.94 ± 0.20 mm, while *Klebsiella pneumoniae* exhibited a slightly higher inhibition zone of 3.92 ± 0.12 mm. *Escherichia coli* showed the lowest inhibition among responsive strains, with an inhibition zone diameter of 1.26 ± 0.21 mm.

**Figure 10:**
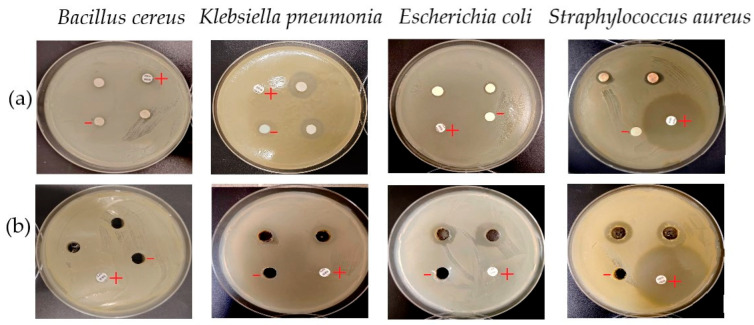
Antibacterial activity of the biosynthesized silver nanoparticles against selected bacterial strains assessed by diffusion methods. (**a**) Disc diffusion method; (**b**) agar well diffusion method. (+) Positive control (penicillin); (−) negative control.

In contrast, the AWD method revealed generally larger zones of inhibition. *E. coli* exhibited the highest zone at5.86 ± 0.51 mm, followed by *S. aureus* at 5.12 ± 0.39 mm and *K. pneumoniae* at 3.64 ± 0.33 mm. These results clearly show that the AWD method provided a more sensitive assessment of the AgNPs’ antimicrobial potential. No antibacterial activity was observed against *Bacillus cereus* with either method (Table 4 and Figure 10).

## 3. Discussion

The phytochemical analysis of *Circaea lutetiana* extracts demonstrated a clear influence of the solvent type on extraction efficiency. The ethanol extract yielded significantly higher amounts of phenolic and flavonoid compounds, with a 15.4 ± 0.6% extraction yield, total phenolic content (TPC) of 89.16 ± 1.15 mg GAEs/g DM, and total flavonoid content (TFC) of 8.06 ± 0.32 mg QEs/g DM. In contrast, the ethyl acetate extract showed substantially lower values, with a TPC of 14.23 ± 0.85 mg GAEs/g DM and a TFC of 7.24 ± 0.18 mg QEs/g DM (Table 1). This is in line with the polarity-based extraction principle, whereby polar solvents like ethanol are more efficient atsolubilizing polyphenols and related metabolites [34].

The HPLC-UV-ESI/MS analysis further substantiated this difference by confirming the presence of multiple phenolic acids, including gallic acid, caffeic acid, and chlorogenic acid, in the ethanol extract, while the ethyl acetate extract selectively contained fewer non-glycosylated flavonoids such as apigenin (Table 2). Gallic acid, the most abundant compound (15.1 mg/g in the ethanol extract), was present at a notably lower level in the ethyl acetate fraction, reinforcing ethanol’s superiority for extracting polar phytoconstituents. These results are consistent with earlier studies by Granica et al. [10] and Granica and Kiss [12], which also highlighted the abundance of polyphenolic compounds in *C. lutetiana*. The presence of phenolic compoundssuggests promising medicinal potential due to their well-documented biological activities. Although these compounds are widely recognized for their antioxidant properties, recent studies increasingly highlight their antimicrobial capabilities. Chlorogenic acid, for example, disrupts bacterial membranes and induces cytoplasmic leakage, leading to cell death [36]. Caffeic acid also shows antibacterial effects, especially against *Staphylococcus aureus*, and may enhance the efficacy of conventional antibiotics [37]. Similarly, gallic acid interferes with microbial enzymes and promotes membrane disruption, contributing to its antibacterial action [38,39,40].

Given the established bioactivity of these molecules—particularly their antioxidant and antimicrobial properties—the ethanol extract was deemed more suitable for downstream green synthesis applications. The choice of ethanol also aligns with green chemistry principles due to its biodegradability and lower toxicity compared to organic solvents like ethyl acetate [41,42]. The decision to use the ethanol extract of *C. lutetiana* for nanoparticle synthesis was further justified by its enriched composition of phenolic compounds, which act as both reducing and capping agents.

The synthesis of silver nanoparticles (AgNPs) was visually evident through a distinct color change from green to brown and further supported by UV–Vis spectroscopy, which showed a sharp surface plasmon resonance (SPR) peak at 408 nm (Figure 2), consistent with literature reports, where silver nanoparticles synthesized using various plant extracts, including *Eucalyptus globulus* and *Oxalis griffithii*, exhibited SPR peaks around this wavelength [34,35].

**Figure 11:**
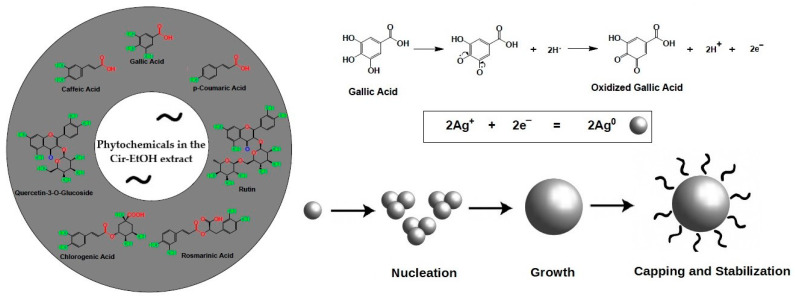
Proposed mechanism for the green synthesis of AgNPs using the *C. lutetiana* extract.

A proposed mechanism for the formation of silver nanoparticles is illustrated in the schematic diagram (Figure 11), which highlights gallic acid as the major reducing and stabilizing agent in the extract, based on the HPLC analysis. This phenolic compound plays a crucial role in the reduction of silver ions (Ag^+^) to metallic silver (Ag^0^), initiating the formation of nanoparticles. Gallic acid, like other phenolic compounds, contains hydroxyl groups capable of donating electrons, which reduce Ag^+^ to Ag^0^ [43]. Furthermore, the oxidized form of gallic acid acts as a stabilizing agent, capping the nascent nanoparticles and preventing their aggregation. While gallic acid was highlighted due to its prominent role in this synthesis, other phenolic compounds in the extract also contribute to the reduction and stabilization process.

The role of phenolic compounds in nanoparticle synthesis is widely recognized. Studies by Mikhailova and colleagues have shown that plant-based phenolics are crucial in reducing metal ions and in stabilizing the resulting nanoparticles [44]. This is in line with our findings, where the rich phenolic content of *Circaea lutetiana*, especially gallic acid, acts dually by reducing Ag^+^ to metallic Ag^0^ and stabilizing the nanoparticles through electrostatic and steric effects.

FTIR analysis underscores the participation of multiple phytochemical functional groups in both the green synthesis and stabilization of silver nanoparticles (AgNPs). The presence of O–H and phenolic groups suggests that polyphenols and flavonoids in the *C. lutetiana* extract actively participated in the reduction of Ag^+^ to Ag^0^, as well as in the stabilization of the resulting nanoparticles. Aliphatic C–H and aromatic C=C stretching bands further imply the contributions of alkyl chains and aromatic compounds, such as flavonoids, in the capping of nanoparticles [45,46]. The observed C–O stretching vibrations confirm the roles of alcohols, esters, and polysaccharides in enhancing colloidal stability. Notably, the weak signal at ~715 cm^−1^ is indicative of possible interactions between silver and bioorganic ligands, supporting the hypothesis of surface coordination between AgNPs and plant-derived compounds [47,48,49].

The comparison of the FTIR spectra of the plant extract and AgNPs revealed slight peak shifts, suggesting chemical interactions between silver and the extract’s functional groups. This aligns with previous studies utilizing *Origanum vulgare* and *Lavandula angustifolia*, which also reported similar spectral changes indicative of nanoparticle formation through green synthesis mechanisms [19,21].

The XPS results validate the successful reduction of Ag^+^ to Ag^0^ during green synthesis using the *C. lutetiana* extract. The Ag 3d peaks are consistent with the findings from earlier research involving silver nanoparticles synthesized through plant-based green methods, such as those using *Mentha arvensis* and *Acacia ehrenbergiana*, which reported comparable binding energies for Ag^0^ in the 368.0–375.0 eV range [50,51,52], confirming the nanoparticle’s metallic nature. The C 1s spectrum reveals signals associated with polyphenols, flavonoids, and carbohydrate residues on the surface of AgNPs, supporting their dual functions as reducing and stabilizing agents—an observation that aligns with the FTIR results. Similar carbon-based functional group patterns have been observed in AgNPs synthesized with *Moringa oleifera* and gallnut extracts [53,54]. The O 1s peak further supports the presence of oxygen-containing functional groups and excludes the significant formation of Ag_2_O, as no shoulder or peak at ~530 eV was found. This indicates that most of the oxygen present is organically bound rather than oxidized silver. Overall, the XPS results complement the FT-IR analysis and reinforce the idea that phytochemical constituents within the extract play key roles in driving both the reduction of silver ions and the stabilization of the resulting nanoparticles.

The XRD data demonstrate that the synthesized AgNPs are crystalline and free of significant impurities, as no additional peaks were observed. This observation is consistent with findings from other research on silver nanoparticles produced through green synthesis. For example, Nidhi Sahu et al. synthesized AgNPs using a *Cynodon dactylon* leaf extract and reported similar diffraction peaks at (111), (200), and (220), confirming the fcc structure and high crystallinity [55]. Likewise, Ruban P. et al. reported AgNPs synthesized from a *Themeda quadrivalvis* extract, with a crystallite size range also falling within 20–25 nm [56]. Additionally, Shalaby et al. found comparable XRD results using a *Zingiber officinale* extract, emphasizing the absence of impurity peaks and the formation of phase-pure nanoparticles smaller than 25 nm [57]. These comparisons further validate our findings and support the reproducibility and reliability of the *C. lutetiana* extract as an efficient agent for the green synthesis of crystalline AgNPs.

The observed hydrodynamic size difference between intensity and number-based distributions reflects the presence of both small nanoparticles and larger aggregates—a common feature in green synthesis due to the variability in phytochemical binding and reduction rates [20,21]. Compared to TEM, DLS often overestimates the particle size, as it includes solvation layers and surface-bound molecules [58]. The PDI of 0.529 confirms moderate polydispersity, aligning with prior reports on greenlysynthesized AgNPs where heterogeneous plant compounds influence particle growth and aggregation. The zeta potential values (−25.62 to −28.19 mV) indicate that the AgNPs exhibit moderate colloidal stability. Although these values fall slightly short of the ±30 mV threshold for high stability, they are consistent with similar studies using *Origanum vulgare* and *Mussaenda frondosa* extracts [19,59]. Furthermore, the FTIR analysis supports the presence of hydroxyl, carbonyl, and ether groups, which aid in nanoparticle stabilization through electrostatic repulsion and steric hindrance [21,44].

The discrepancy between particle sizes obtained via transmission electron microscopy and dynamic light scattering measurements, specifically 3.84 ± 0.62 nm by TEM and 192 nm by intensity-based DLS, strongly indicates nanoparticle aggregation in the colloidal suspension. This variation is due to the difference in measurement principles: TEM determines the true metallic core size of dried, individual nanoparticles, while DLS captures the hydrodynamic diameter, which also accounts for solvation layers and phytochemicals attached to the particle surface. As a result, DLS often reports significantly larger sizes, especially in the presence of agglomerates. Such a disparity is well documented in nanoparticle research and is often attributed to van der Waals attractions, limited electrostatic repulsion, or unfavorable solvent conditions [58].

TEM images showed that the biosynthesized silver nanoparticles were evenly distributed, predominantly spherical, and displayed little to no aggregation. The nanoparticles measured between 2.70 and 4.95 nm in size, with an average diameter of 3.84 ± 0.62 nm, confirming the formation of ultrafine particles. These nanoscale dimensions are consistent with previous studies using green synthesis methods. For instance, AgNPs synthesized with a *Zingiber officinale* extract showed an average size of approximately 3.1 nm, attributed to active biomolecules in the extract acting as reducing and stabilizing agents [57]. Similarly, Abdelghany et al. [19] summarized that AgNPs synthesized via plant and fungal extracts typically fall within the 5–30 nm range, with spherical particles dominating. Their review emphasized that particles sized 1–10 nm display enhanced biological activity due to higher surface-to-volume ratios. As such, the <5 nm size of our synthesized nanoparticles positions them within this favorable range. Additionally, Ramya and Subapriya reported that controlled green synthesis using natural phytochemicals can yield well-dispersed nanoparticles below 10 nm with improved functional properties [60].

The SEM images confirmed effective capping and shape control, likely attributable to flavonoids and phenolic acids in the *C. lutetiana* extract [19,20]. The discrepancy between TEM and SEM sizes is common: TEM reflects the metallic core, whereas SEM includes surface coating and minor aggregation [61].

Finally, the elemental analysis (EDX) validated the formation of elemental silver, while the presence of carbon and oxygen further supports the incorporation of phytochemicals as reducing and stabilizing agents [19,62]. EDX confirmed the even coating of phytochemicals over the nanoparticles, which helps prevent aggregation and enhances dispersion stability. These findings complement the FTIR and XPS results and confirm the multifunctional roles of plant-based bioactives in green nanoparticle synthesis.

The silver nanoparticles (AgNPs) synthesized in this study retain bioactive phytochemicals, such as flavonoids and phenolic acids, from the *C. lutetiana* extract, which may enhance their antibacterial activity in addition to providing stabilization. These plant-derived compounds, particularly gallic acid, rutin, and quercetin-3-glucoside, act not only as reducing agents during synthesis but may also participate directly in antimicrobial action through membrane disruption and interference with bacterial metabolism [63].

In comparative terms, the ethanolic extract of *C. lutetiana* displayed only weak antibacterial activity, with minimal inhibition zones in the disc diffusion method (DDM). This is likely due to the limited diffusion capacity of complex phytochemical mixtures in agar, as well as the generally milder bacteriostatic effects of antioxidant-rich profiles [14]. In contrast, the ethyl acetate extract demonstrated no inhibitory activity at all, which can be attributed to its lower polarity and consequently lower contents of active phenolic compounds.

Upon green synthesis, however, the antibacterial activity of AgNPs significantly improved, especially in agar well diffusion (AWD) tests. The nanoparticles produced clear inhibition zones against *E. coli* (5.86 mm), *S. aureus* (5.12 mm), and *K. pneumoniae* (3.64 mm), in line with the findings of Okafor et al. [16] and Abdelghany et al. [20]. The AWD method proved more sensitive than the DDM, primarily because the well system allows direct contact between the nanoparticle suspension and the bacterial lawn, leading to better diffusion and local accumulation of nanoparticles at the site of inoculation.

Other studies using *Azadirachta indica* extracts have reported larger inhibition zones, but in our study, comparable effects were achieved using smaller AgNPs volumes [64]. This suggests that the unique phytochemical composition of *C. lutetiana* may enhance nanoparticle activity even at lower concentrations.

One of the possible mechanisms of antibacterial action may involve the generation of reactive oxygen species (ROS), disruption of cell membranes, and interference with DNA and protein synthesis [65,66,67]. Additionally, Gram-negative bacteria may be more susceptible due to their thinner and more permeable cell walls [68].

Collectively, these findings support the use of *C. lutetiana* as a promising plant source for the eco-friendly synthesis of bioactive silver nanoparticles and highlight the synergistic roles of its phytochemicals in both nanoparticle formation and antimicrobial action.

## 4. Materials and Methods

### 4.1. Reagents, Chemicals, and Standards

A variety of chemical reagents were used in spectrophotometric analyses for evaluating phytochemicals, including Folin–Ciocalteu reagent (2 M), gallic acid (≥98%), quercetin (≥95%), trichloroacetic acid (≥99%), sodium carbonate (≥99.5%), and anhydrous aluminum chloride (≥99.99%). These substances were mainly applied to assess the total phenolic content (TPC) and total flavonoid content (TFC) in the extracts.

For HPLC-UV-ESI/MS profiling, a set of 17 standard phenolic compounds was chosen, comprising gallic acid (≥99%), caffeic acid (≥98%), chlorogenic acid (≥95%), ferulic acid (≥99%), rosmarinic acid (≥98%), catechin (≥98%), epicatechin (≥98%), naringin (≥98%), rutin (≥94%), luteolin-7-O-glucoside (≥98%), luteolin (≥98%), quercetin (≥95%), apigenin (≥95%), kaempferol (≥97%), dihydroquercetin (≥90%), myricetin (≥96%), and naringenin (≥95%). All reference standards and analytical-grade reagents were obtained from Sigma-Aldrich (Burlington, MA, USA).

Analytically pure organic solvents, including methanol, ethanol, ethyl acetate, and dimethyl sulfoxide (DMSO), were sourced from local suppliers. For chromatographic separation, HPLC-grade acetonitrile (≥99.9%) and formic acid (99–100%) were supplied by Sigma-Aldrich (Briare, France) and VWR Chemicals (Briare, France), respectively. Ultrapure water used throughout the analyses was produced using a Milli-Q purification system (Millipore, Guyancourt, France).

For measurements of the zeta potential and particle size by dynamic light scattering (DLS), a 0.1 M potassium nitrate (KNO_3_) solution was used as the dispersion medium to ensure reproducibility and ionic stability during nanoparticle characterization.

### 4.2. Plant MaterialCollection and Extraction

Aerial portions of *Circaea lutetiana* L. were collected in August 2024 from Bayanaul National Park (Pavlodar region, Kazakhstan) under the guidance of the Institute of Botany and Phytointroduction (IBP). Taxonomic identification was conducted by specialists at the IBP herbarium, and a voucher sample (IBP 5829) was deposited in their official archive. Following collection, the plant parts were rinsed thoroughly with distilled water to eliminate residual contaminants. Subsequently, the material was dried naturally in a shaded, well-ventilated environment until complete dehydration. The dried plant mass was then ground into a fine powder using an electric microgrinder (110 V, 1400 rpm) and kept at −20 °C in sealed containers until further use.

To isolate bioactive compounds, Soxhlet extraction was applied to the powdered plant samples. For each extraction run, 20 g of the powdered material wassubjected to solvent extraction with 200 mL of either ethanol or ethyl acetate for 5 h. Upon completion, the extract solutions were filtered through Whatman paper to remove solid residues, and the solvents were evaporated under reduced pressure at 40 °C using a rotary evaporator (IKA RV10 auto V-C, Staufen, Germany). The obtained semi-solid extracts were left to dry at ambient temperature until complete solidification. The percentage yield (W, %) of the extract was calculated using the following formula:

W (%) = (m_1_/m_0_) × 100

where m_1_ represents the dry mass of the obtained extract and m_0_ denotes the initial dry mass of the plant material used.

### 4.3. Phytochemical Analysis

#### 4.3.1. Total Phenolic and Flavonoid Contents (TPC and TFC)

The quantification of the total phenolic content (TPC) was performed via a modified Folin–Ciocalteu colorimetric method based on the standard procedure outlined by Singleton and Rossi [69]. Each plant extract was first dissolved in methanol to obtain a stock solution at a concentration of 1000 µg/mL. A 0.25 mL aliquot of this solution was combined with 1 mL of a tenfold diluted Folin–Ciocalteu reagent, followed by the addition of 0.75 mL of 1% sodium carbonate. The reaction mixture was incubatedin the dark at ambient temperature for two hours. After the incubation, the absorbance was measured at 760 nm using a DR3900 spectrophotometer. A standard calibration curve was prepared using gallic acid at varying concentrations (25, 50, 75, and 100 µg/mL). The TPC results were expressed as milligrams of gallic acid equivalents per gram of dried extract (mg GAEs/g DM).

The total flavonoid content (TFC) was assessed through the aluminum chloride colorimetric assay. Briefly, 1 mL of the extract solution (1000 µg/mL in methanol) was mixed with an equal volume of 2% AlCl_3_ in methanol. This mixture was left to react in the dark at room temperature for 15 min. The resulting absorbance was recorded at 430 nm. To quantify the TFC, a standard curve was constructed using quercetin solutions at concentrations of 6.25, 12.5, 25, and 50 µg/mL. The flavonoid content was then calculated and expressed in terms of milligrams of quercetin equivalents per gram of dry extract (mg QEs/g DM) [63].

#### 4.3.2. Analysis of Phenolic Compounds Using High-Performance Liquid Chromatography Coupled with UV and Electrospray Ionization Mass Spectrometry

The extract was analyzed using high-performance liquid chromatography coupled with ultraviolet detection and electrospray ionization mass spectrometry (HPLC-UV-ESI/MS), employing the same instrumentation parameters and analytical procedure as outlined in our earlier work involving *Chamaenerion latifolium* [63].

### 4.4. Green Synthesis of Silver Nanoparticles

**Figure 12:**
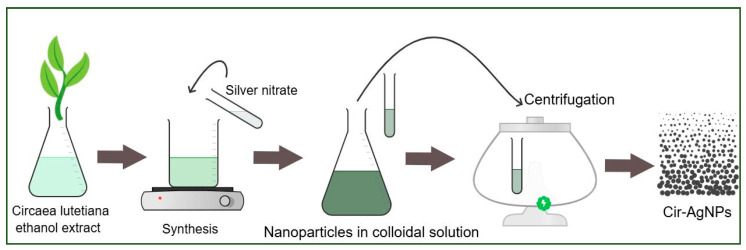
Green synthesis of AgNPs using the *C. lutetiana* ethanol extract and silver nitrate, followed by centrifugation.

The green synthesis of silver nanoparticles (AgNPs) was carried out using the ethanol extract of *C. lutetiana* (Figure 12). For this, 10 mL of the ethanol plant extract was added to 10 mL of an aqueous silver nitrate (AgNO_3_) solution at a concentration of 3 mM, maintaining a 1:1 ratio. The mixture was stirred continuously and heated at 60 °C for 60 min to facilitate the reduction of Ag^+^ ions. A gradual color change from dark green to dark brown indicated the formation of AgNPs.

After the reaction, the suspension was allowed to stand at room temperature for 24 h to ensure complete nanoparticle formation. The synthesized nanoparticles were collected by centrifugation at 10,000 rpm for 15 min and washed several times with distilled water and ethanol to remove any residual impurities. The purified AgNPs were then dried at 60 °C and stored in a desiccators for further analysis.

### 4.5. Characterization of Silver Nanoparticles

To thoroughly investigate the physicochemical characteristics of the biosynthesized silver nanoparticles (AgNPs), multiple complementary analytical techniques were applied.

The crystalline structure of the AgNPs was analyzed via X-ray diffraction (XRD) using a Rigaku SmartLab diffractometer (Cedar Park, TX, USA) to confirm their phase identity and crystallinity [70]. The morphological features and surface integrity of the nanoparticles were further visualized through scanning electron microscopy (SEM) utilizing a Zeiss Crossbeam 540 microscope (Jena, Germany). To gain deeper insights into internal structure and particle size dispersion, transmission electron microscopy (TEM) was employed using a JEOL JEM–1400 Plus instrument (JEOL Ltd, Tokyo, Japan) [71].

To identify the functional groups involved in the nanoparticle synthesis and stabilization processes, Fouriertransform infrared (FT-IR) spectroscopy was conducted on both the plant extract and AgNP samples. Measurements were performed in attenuated total reflectance (ATR) mode using a Nicolet™ 6700 FT-IR spectrometer (Thermo Scientific, Waltham, MA, USA) equipped with a diamond ATR crystal. Spectra were collected within the 4000–500 cm^−1^ range at a 4 cm^−1^ resolution, averaging 32 scans per sample. Data acquisition and spectral interpretation were performed using Omnic 5.2 software [63]. This method enabled the identification of specific chemical groups associated with nanoparticle formation and stabilization.

The elemental analysis and atomic distribution within the nanoparticles were examined using energy-dispersive X-ray spectroscopy (EDS). Furthermore, to analyze the oxidation states and electronic structures of the constituent elements, X-ray photoelectron spectroscopy (XPS) was carried out with a NEXSA spectrometer (Thermo Scientific, Waltham, MA, USA) [72].

The colloidal behavior and particle size distribution of the silver nanoparticles in aqueous medium were evaluated through a dynamic light scattering (DLS) analysis, along with zeta potential measurements. These assessments were carried out using a NanoBrook Omni instrument (Brookhaven Instruments, USA) to determine the dispersion stability and surface charge characteristics of the nanoparticles [73].

To explore the optical behavior of the synthesized AgNPs, UV–visible spectroscopy was employed using a Thermo Scientific Evolution 300 spectrophotometer. This technique enabled the recording of absorption spectra, confirming nanoparticle formation and the presence of surface plasmon resonance features typical of metallic silver nanoparticles [74].

### 4.6. Evaluation of Antimicrobial Activity

Evaluation of Antimicrobial Activity: The antimicrobial activity of the *Circaea lutetiana* extracts and biosynthesized silver nanoparticles (AgNPs) was evaluated against selected pathogenic bacterial strains using agar diffusion methods [75].

Test Microorganisms: The following Gram-positive and Gram-negative bacterial strains were used: *Staphylococcus aureus, Bacillus cereus, Escherichia coli*, and *Klebsiella pneumoniae*. All strains were obtained from the Republican Microbial Culture Collection (Astana, Kazakhstan).

Preparation of Inoculum: Bacterial cultures were prepared by transferring a single colony from nutrient agar into 3 mL of sterile meat–peptone broth. The suspensions were incubated at 37 °C for 24 h. Prior to inoculation, the bacterial density was adjusted to 3 × 10^8^ CFUs/mL to match a turbidity equivalent to McFarland standard 1.0 IU.

Disc Diffusion Method: The ethanolic and ethyl acetate extracts of *C. lutetiana*, as well as AgNPs synthesized from the ethanolic extract, were prepared in DMSO at a final concentration of 0.1 mg/mL. Sterile paper discs (6 mm diameter) were impregnated with 100 μL of each test solution and allowed to air dry under aseptic conditions. The discs were then placed on nutrient agar plates pre-inoculated with the bacterial suspension using sterile swabs.

Penicillin (10 μg/disc) served as a positive control, while discs containing 100 μL of DMSO were used as a negative control. The plates were incubated at 37 °C for 18–24 h, after which the inhibition zone diameters (IZDs) were measured in millimeters using digital calipers.

Well Diffusion Method: To assess the antimicrobial activity of AgNPs using an additional approach, the well diffusion method was applied. After inoculating the agar surface with a bacterial suspension, wells with a diameter of 7.5 mm were aseptically punched into the agar. Into each well, 100 μL of the corresponding test bacterial suspension was added. Silver nanoparticles were prepared as a colloidal suspension at a concentration of 0.1 mg/mL, and 100 μL of this suspension in sterile saline solution was then introduced into the wells.

For the negative control, the sterile saline solution (100 μL) was used in place of the AgNP suspension. All plates were incubated at 37 °C for 24 h. Following the incubation, the antibacterial activity was assessed by measuring the diameter of the inhibition zones. The clear zone was measured from the edge of the well to the edge of the inhibition area on both opposite sides, and the values were summed.

The sensitivity interpretation was as follows: ≤1.0 mm—resistant (no sensitivity); 1.1–4.9 mm—low sensitivity; 5.0–8.9 mm—moderate sensitivity; and ≥9.0 mm—high sensitivity. All experiments were carried out in triplicate to ensure accuracy and reproducibility.

### 4.7. Statistical Analysis

All quantitative results were subjected to statistical analysis using GraphPad Prism software (version 5.0; GraphPad Software Inc., La Jolla, CA, USA). To compare group means, one-way analysis of variance (ANOVA) was applied, followed by Duncan’s multiple range test to identify statistically significant differences, considering a significance threshold of *p* < 0.05. The data are presented as the means ± standard deviations (SDs) calculated from three separate experimental replicates.

## 5. Conclusions

This study highlights the potential of *Circaea lutetiana* as a sustainable botanical source for the green synthesis of silver nanoparticles (AgNPs). Phytochemical profiling of ethanol and ethyl acetate extracts revealed a notable abundance of phenolic acids and flavonoid glycosides in the ethanol extract, including gallic acid, caffeic acid, and rutin—compounds known to possess both reducing and stabilizing capabilities in nanoparticle formation. Guided by this chemical richness, the ethanol extract was employed in the green synthesis of AgNPs, producing uniformly dispersed, spherical nanoparticles with an average size of 3.84 ± 0.62 nm and a well-defined crystalline structure.

Comprehensive characterization using UV–Vis, FT-IR, TEM, SEM-EDX, XRD, DLS, and XPS confirmed the successful formation and stability of the nanoparticles. The synthesized AgNPs exhibited significant antibacterial activity, particularly when tested via agar well diffusion, outperforming the crude plant extracts, which displayed only weak or no inhibitory effects. These findings confirm that converting plant-based phytochemicals into nanostructures can significantly enhance their biological activity.

Importantly, this is the first report demonstrating the biosynthesis of AgNPs from *C. lutetiana*, establishing its value not only as a phytochemical reservoir but also as a practical tool in eco-friendly nanotechnology. The combined antimicrobial effects of silver ions and plant-derived capping agents suggest potential applications in biomedicine, particularly for antimicrobial coatings, topical formulations, and possibly as adjuncts to antibiotic therapies. Future studies may explore in vivo efficacy, cytotoxicity, and broader antimicrobial spectra, opening pathways for the clinical and pharmaceutical development of *C. lutetiana*-based nanomaterials.

## Figures and Tables

**Table 1:** Total phenolic and flavonoid compounds in *C. lutetiana* extracts.

Extract	TPC (mg GAEs/g DM) *	TFC (mg QEs/g DM) *
Cir-EtOAc	14.23 ± 0.85	7.24 ± 0.18
Cir-EtOH	89.16 ± 1.15	8.06 ± 0.32

* QEs stand for quercetin equivalents, GAEsstand for gallic acid equivalents, and DM stands for dry matter. The statistical analysis using ANOVA showed significant differences at *p* < 0.05. All values are presented as the means ± standard deviations from three replicates of a single independent experiment.

**Table 2:** Phenolic profiles of the EtOH and EtOAc extracts of the *C. lutetiana* aerial parts, as determined by HPLC-UV-ESI/MS.

Peak No.	RT (min)	[M+H]^−^ (*m*/*z*)	Identified Metabolite	Subclass	Molecular Formula	Cir-EtOH *	Cir-EtOAc *
1	3.92	179	Caffeic acid	Phenolic acid	C_9_H_8_O_4_	9.80	0.34
2	5.09	169	Gallic acid	Phenolic acid	C_7_H_6_O_5_	15.1	3.40
3	13.47	353	Chlorogenic acid	Phenolic acid (glycoside)	C_16_H_18_O_9_	10.5	_
4	19.17	609	Rutin	Flavonoid (glycoside)	C_27_H_30_O_16_	1.20	_
5	20.03	463	Quercetin 3-glucoside	Flavonoid (glycoside)	C_21_H_19_O_12_	0.10	0.03
6	20.35	301	Not identified	–	–	–	–
7	20.79	163	*p*-Coumaric acid	Phenolic acid	C_9_H_8_O_3_	14.6	–
8	21.93	193	Ferulic acid	Phenolic acid	C_10_H_10_O_4_	–	0.10
9	22.70	359	Rosmarinic acid	Phenolic acid	C_18_H_16_O_8_	0.29	–
10	35.36	269	Apigenin	Flavonoid	C_15_H_10_O_5_	–	0.02

* Phenolic content in DM (mg/g).

**Table 3:** Antibacterial activity of *C. lutetiana* extracts (disc diffusion method).

Microorganisms Tested	Gram Type	Extract		Positive Control
		Cir-EtOH	Cir-EtoAc	Penicillin, IZD, mm
*Bacillus cereus*	Gram +	NA	NA	NA
*Straphylococcus aureus*	Gram +	0.59 ± 0.18 ^a^	NA	13.08 ± 0.80
*Escherichia coli*	Gram −	1.07 ± 0.24 ^a^	NA	NA
*Klebsiella pneumonia*	Gram −	2.54 ± 0.36 ^b^	NA	NA

IZD: inhibition zone diameter. NA: no activity. The results presented as the means ± SDs. Different letters in the column indicate statistically significant differences among bacterial strains according to Duncan’s multiple range tests (*p* < 0.05). Note: Antagonistic activity was classified as follows: no activity for inhibition zones up to 1.0 mm, low activity for zones between 1.1 and 4.9 mm, moderate activity for 5.0 to 8.9 mm, and high activity for zones measuring 9.0 mm or more.

**Table 4:** Antibacterial activity of AgNPs (disc and well diffusion methods).

Microorganisms Tested	Gram Type	AgNPs		Positive Control	
		DDM	AWD	Penicillin, IZD, mm(DDM)	Penicillin, IZD, mm (AWD)
*Bacillus cereus*	Gram +	NA	NA	NA	NA
*Straphylococcusaureus*	Gram +	2.94 ± 0.20 ^b^	5.12 ± 0.39 ^a^	16.15 ± 0.87	15.86 ± 0.73
*Escherichia coli*	Gram −	1.26 ± 0.21 ^a^	5.86 ± 0.51 ^a^	NA	NA
*Klebsiella pneumonia*	Gram −	3.92 ± 0.12 ^c^	3.64 ± 0.33 ^b^	NA	NA

IZD: inhibition zone diameter. NA: no activity. The results presented as the means ± SDs. Different letters within the same column indicate statistically significant differences among bacterial strains according to Duncan’s multiple range tests (*p* < 0.05). Note: The antagonistic activity of the studied cultures is considered zero when the width of the zone of no growth is up to 1.0 mm, lowat1.1–4.9 mm, mediumat 5.0–8.9 mm, and highat 9.0 mm or more. DDM—disk diffusion method; AWD—agar well diffusion method.

## Data Availability

The data are contained within the article and Supplementary Material.

## Supplementary Materials

The following supporting information can be downloaded at https://www.mdpi.com/article/10.3390/ijms26125505/s1.

## Abbreviations

The following abbreviations are used in this manuscript:

ANOVA	Analysis of Variance
AWD	Agar Well Diffusion
CFU	Colony Forming Units
DDM	Disc Diffusion Method
DLS	Dynamic Light Scattering
DM	Dry Mass
DMSO	Dimethyl Sulfoxide
DNA	Deoxyribonucleic Acid
DPPH	2,2-Diphenyl-1-picrylhydrazyl
EDX	Energy Dispersive X-ray Spectroscopy
FT-IR/FTIR	Fourier Transform Infrared Spectroscopy
GAE	Gallic Acid Equivalent
GC-MS	Gas Chromatography–Mass Spectrometry
HPLC-UV-ESI	HPLC with UV detection and Electrospray Ionization
IBP	Institute of Botany and Phytointroduction
ICP-MS	Inductively Coupled Plasma–Mass Spectrometry
IZD	Inhibition Zone Diameter
JCPDS	Joint Committee on Powder Diffraction Standards
NPs	Nanoparticles
PDI	Polydispersity Index
QE	Quercetin Equivalent
ROS	Reactive Oxygen Species
SD	Standard Deviation
SEM-EDX	SEM coupled with Energy Dispersive X-ray Analysis
SPR	Surface Plasmon Resonance
TEM	Transmission Electron Microscopy
TFC	Total Flavonoid Content
TPC	Total Phenolic Content
UV	Ultraviolet
XPS	X-ray Photoelectron Spectroscopy
XRD	X-ray Diffraction
