# Supplementary material for: Green Synthesis of Silver Nanoparticles Using Circaea lutetiana Ethanolic Extract: Phytochemical Profiling, Characterization, and Antimicrobial Evaluation
Source: Int J Mol Sci. 2025 Jun 8;26(12):5505. doi: 10.3390/ijms26125505 (PMC12193652; doi:10.3390/ijms26125505)
Supplement: Supplementary file 1 [file ijms-26-05505-s001.zip › Supplementary Materials.pdf]

Supplementary Data

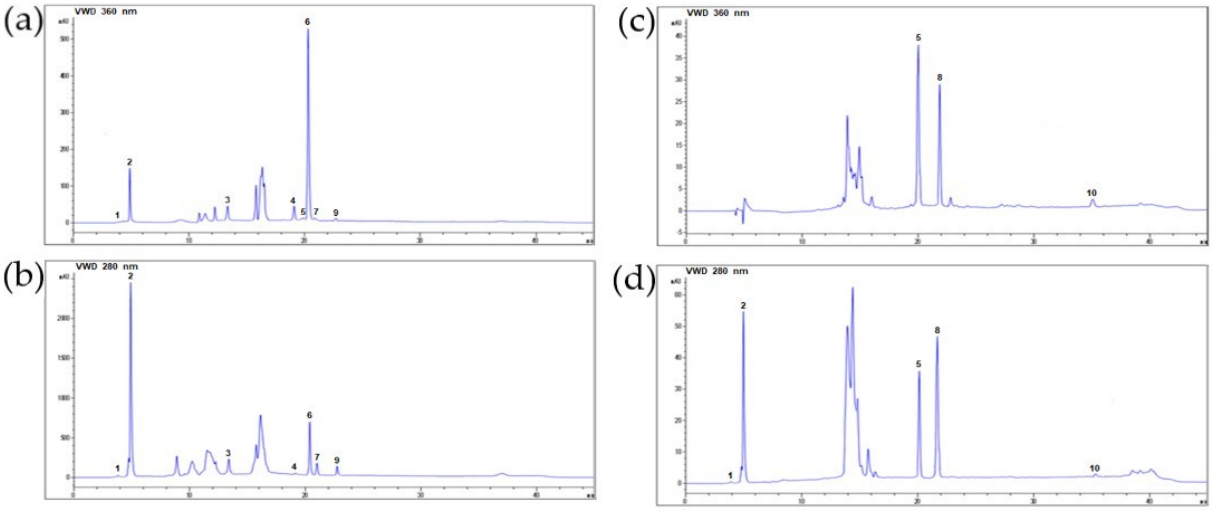

Figure S1. UV chromatograms of *C. lutetiana* extracts recorded using HPLC-UV-ESI/MS: ethanol extract at 360 nm (a) and 280 nm (b), and ethyl acetate extract at 360 nm (c) and 280 nm (d).
